# Hygiene1Read by title at the annual meeting of the New York State Veterinary Medical Association, September 6, 1895.

**Published:** 1895-11

**Authors:** Wilson Huff

**Affiliations:** Rome, N. Y.


### HYGIENE.[1]

BY DR. WILSON HUFF,
ROME, N. Y.

HYGIENE is the art of preserving health; that is, of obtaining the most perfect action of body and mind as long a period as is consistent with the laws of life. In other words, it aims at rendering growth more perfect, decay less rapid, life more vigorous, death more remote. This art has been practised from the earliest times. Before Hippocrates there were treatises on hygiene which that great master evidently embodied in his incomparable works. It was then based on what we should now call empirical rules, simply on observation of what seemed good or bad for health. Very early, indeed, the effects of diet and exercise were carefully noticed and were considered the basis of hygiene. Hippocrates appears to have had a clear con-

[1] Read by title at the annual meeting of the New York State Veterinary Medical Association, September 6, 1895.

ception of the relations between the amount of food taken and of the mechanical energy produced by it; at least he is extremely careful in pointing out that there must be an exact balance between food and exercise, and that disease results from excess either way. The effects on health of different kinds of air, of water, and, to some extent, of soils, were also considered at a very early date, though naturally the ignorance of chemistry prevented any great advance in this direction. Hippocrates summed up the existing knowledge of his time on the six articles which, in after days, received the absurd name of the non-naturals. The six articles were air, food, exercise, rest, sleep, evacuations.

With the exception of the attempts of the alchemists and of the chemical physicians to discover some agent or drug which might increase or strengthen the principle of life, the practice of hygiene remained within the same limits until physiology (the knowledge of the laws of life) began to be studied. Hygiene then began to acquire a scientific basis, still retaining its empirical foundation drawn from observation. It commenced to apply the discoveries of physiology to the improvement of health and to test the value of its own rules by this new light. It is now gradually becoming an art based on the science of physiology, with whose progress its future is identified.

But the art of hygiene has, at present, still another object. If we had a perfect knowledge of the laws of life, and could practically apply this knowledge in a perfect system of hygienic rules, disease would be impossible; but at present disease exists in a thousand forms, and the human race languishes and at times almost perishes under the grievous yoke. The study of the causes of disease is strictly a part of physiology, but it can only be carried out by the practical physician, since an accurate identification of the disease is the first necessary step in the investigation of causes. The causes being investigated, the art of hygiene then comes in to form rules which may prevent the causes, or render the frame more fitted to bear them; and as in the former case it was the exponent of physiology, in this case it becomes the servant of the pathologist. In relation to the natural conditions which surround us and which are essential for life, such as the air, water, and food, the source of all bodily and mental acts, the soil we move on and the sun which gives warmth; light, etc., in fact, relation to Nature at large; in our capacity as an independent being having within ourselves sources of action, in thoughts, feelings, desires, personal habits, all of which affect health, and which require self-regulation and control.

Even now, incomplete as hygiene necessarily is, such a work would, if followed, almost change the face of the world. But, would it be followed? In some cases the rules of hygiene could not be followed however much the individual might desire to do so. For example, pure air is a necessity for health, but, as an individual may have little control over the air which surrounds him and which he must draw into his lungs, he may be powerless to prevent other persons from contaminating his air and thereby striking at the very foundation of his health and happiness. Here, as in so many other cases which desire regulation, the State steps in for the protection of its citizens and enacts laws which shall be binding upon all. Hence, arises what is termed State medicine. There is probably no branch of the medical profession for which all questions with reference to State medicine have more vital interest than the veterinarian. While the medical practitioner is assured of his social position so long as he attends to his practice, it is essential to the veterinarian that his value as an agent upon all questions with regard to contagious animal-disease be recognized by the State. This is not the case in the United States, hence it is that I have thought it well that we should have some idea of what is understood by State medicine. The careful student of the medical institutions of the United States must most certainly admit that there is much rottenness to be rooted out before they can become, not only what they should be, but worthy of so wealthy a country and so energetic and practical a people. In speaking of the medical institutions of the country, you must not think the schools alone are to be the subject for consideration. They form but a unit among many; but, unfortunately for many schools as well as for the people and the nation, it is the most important and fundamental unit in the whole organism. If the foundation is weak the whole superstructure must be in a like condition. Before entering upon the discussion of this, the educational part of our question, it is essential that we come to a clear understanding of what we mean by the medical institutions of a country. To this end it is necessary that we define our ideas of medical science. The ultimate endeavor or purpose of medical science as we understand it to-day is not to educate practitioners in medicine or make doctors alone, but rather subject all disease to the most searching intellectual, physical, chemical, macro- and microscopic analysis possible, in the hope that we may thus be enabled to discover, not only the nature of a given disease, but also the nature of its causes—for all diseases have their internal organismal or predisposing causes, as well as the exciting causes—in order that we may elucidate means by which to prevent the generation of such disease so far as possible in the future. Up to the present time our endeavors in this direction have been almost entirely limited to diseases of an infectious or contagious nature. Our work in the future must also include the so-called sporadic or, as I would term them, common diseases of life, the chief cause of which is to be sought in the utter ignorance of mankind of the laws of life. The bacillus of ignorance plays a much greater *rôle* in the causation of disease than that of phthisis, cholera, or septicæmia, and will be more difficult to combat and expose.

The medical institutions of a country are: First, the schools; second, the boards of health, or its preventive force; third, the practitioners. The science of medicine is the great life-saving service of the world. The medical institutions of this country should be as much a part of its life-saving service as that great body of men that have been so successfully organized along our seaboard and lake coasts.

But no fixed rule can be applied to this, for what is health in one may be disease in another. The predisposing and exciting causes of disease, when existing within the system, are called intrinsic; but when they arise without the system, they are extrinsic, exopathic, or external causes of disease. The most important and generally recognized predisposing causes of disease are: the influence of age, dentition, rickets, canine distemper, strangles. Invasion of parasites is much more common during the earlier period of an animal's life; for example, the *strongylus filaria* of the lamb and its analogue, the *strongylus micrurus* of the calf. The bones are much more liable to disease during youth than middle age; for example, osteoporosis. Again, we find disease of the facial bones, peculiarities of breed and conformation, canker, grease, roaring, etc., the effects of color, and round-chested horses are more liable to become broken-winded.

Experiments have proved that in order to support health and strength it is essential that, in addition to water, food contain at least three classes of conditions, namely: First, nitrogenous food to nourish muscular and other albuminoid tissues; second, hydrocarbons, which supply materials that undergo combustion in the body and assist in the maintenance of animal heat and assimilation of nitrogenous compounds; third, salines, to supply materials for the building up of the solid structures of the body, maintaining them in health, and assisting in the processes of assimilation and elimination, conveying new materials into the system and removing old ones out of it. Milk offers us the best example, as it contains casein, nitrogenous oil and sugar, hydrocarbons, water, and salts. Grass may also be added, as food containing all necessary to support life.

